# Supplementary material for: Resolving the full spectrum of human genome variation using Linked-Reads
Source: Genome Res. 2019 Apr;29(4):635–45. doi: 10.1101/gr.234443.118 (PMC6442396; doi:10.1101/gr.234443.118)
Supplement: Supplemental Material [file supp_gr.234443.118_Supplemental_longranger-2.2.zip › longranger-2.2.tar.bz2!/lr2.2-public/lib/stan/cmdstan/stan/doc/api/html/structstan_1_1math_1_1detail_1_1bounded_3_01_t__y_00_01_t__low_00_01_t__high_00_01_t__result_00_01true_01_4.html]

Stan: stan::math::detail::bounded< T\_y, T\_low, T\_high, T\_result, true > Struct Template Reference


|  |  |
| --- | --- |
|  | Stan  2.5.0  probability, sampling & optimization |


- Main Page
- Related Pages
- Namespaces
- Classes
- Files

- Class List
- Class Index
- Class Hierarchy
- Class Members

All Classes Namespaces Files Functions Variables Typedefs Enumerations Enumerator Friends Macros Pages

- stan
- math
- detail
- bounded< T\_y, T\_low, T\_high, T\_result, true >

Static Public Member Functions |
List of all members

stan::math::detail::bounded< T\_y, T\_low, T\_high, T\_result, true > Struct Template Reference

`#include <check_bounded.hpp>`

|  |  |
| --- | --- |
| Static Public Member Functions | |
| static bool | check (const char \*function, const T\_y &y, const T\_low &low, const T\_high &high, const char \*name, T\_result \*result) |
|  | |

## Detailed Description

### template<typename T\_y, typename T\_low, typename T\_high, typename T\_result> struct stan::math::detail::bounded< T\_y, T\_low, T\_high, T\_result, true >

Definition at line 51 of file check\_bounded.hpp.

## Member Function Documentation

template<typename T\_y , typename T\_low , typename T\_high , typename T\_result >

|  |  |  |  |
| --- | --- | --- | --- |
| static bool stan::math::detail::bounded< T\_y, T\_low, T\_high, T\_result, true >::check | ( | const char \* | *function*, |
|  |  | const T\_y & | *y*, |
|  |  | const T\_low & | *low*, |
|  |  | const T\_high & | *high*, |
|  |  | const char \* | *name*, |
|  |  | T\_result \* | *result* |
|  | ) |  |  |

inlinestatic

Definition at line 52 of file check\_bounded.hpp.

---

The documentation for this struct was generated from the following file:

- src/stan/math/error\_handling/check\_bounded.hpp


---

[ Stan Home Page ]

*© 2011–2014,
Stan Development Team.*
